# A Survey on Transport Management Practices Associated with Injuries and Health Problems in Horses

**DOI:** 10.1371/journal.pone.0162371

**Published:** 2016-09-02

**Authors:** Barbara Padalino, Sharanne L. Raidal, Evelyn Hall, Peter Knight, Pietro Celi, Leo Jeffcott, Gary Muscatello

**Affiliations:** 1 School of Life and Environmental Sciences, Faculty of Veterinary Science, The University of Sydney, Camden, New South Wales, Australia; 2 Department of Veterinary Medicine, The University of Bari, Bari, Italy; 3 School of Animal and Veterinary Sciences, Charles Sturt University, Wagga Wagga, New South Wales, Australia; 4 Discipline of Biomedical Science, School of Medical Sciences, Sydney Medical School, University of Sydney, Sydney, Australia; Cardiff University, UNITED KINGDOM

## Abstract

An online survey was conducted to determine associations between transport management and transport-related injuries and diseases in horses in Australia. The survey was composed of three sections: respondents’ demographic information, transport management strategies or procedures (before, during and after transportation) and transport diseases experienced in the previous two year period. Univariate and multivariate modelling was performed exploring associations between variables (respondents’ details and transport management strategies) and the following transport-related diseases as outcomes: traumatic injuries, diarrhoea, heat stroke, muscular problems, laminitis, transport pneumonia and colic. The survey generated 797 responses. Traumatic injuries were the most common transport-related problem, with a reported incidence of 45.0%. Younger respondents (<40 years old) caring for large numbers of horses (>30 in a week) were more likely to report transport-related injuries. Injury risk was also linked to the use of protections and tranquilizers prior to transport, and checking horses after the journey. Diarrhoea (20.0%) and heat stroke (10.5%) were reported more by amateur than professional horse carers. Increased risk of heat stroke was linked to the restriction of hay and water prior to transportation. Muscular problems (13.0%) appeared to be exacerbated when horse health was not assessed before journey; whilst the risk of laminitis (2.9%) was around three fold greater when post transport recovery strategies were not applied. Associations were made between transport pneumonia (9.2%) and duration of journey, and with activity (horses involved in racing at greater risk). No associations were seen between the incidence of colic (10.3%) and the variables examined. Study findings should be interpreted with caution as they represent participant perceptions and recall. Nevertheless, results support many current recommendations for safe transportation of horses. They also highlight the need to further investigate many of identified management factors to refine existing policies and practices in equine transportation.

## Introduction

The act of transportation has been associated with traumatic injuries and diseases in horses [1]. Traumatic injuries, such as lacerations and contusions, to horses and horse handlers are considered to be the most common problem arising from equine transportation [2]. Results of a survey on horse injuries have shown that, apart from the paddock, the trailer is the second most likely place for horses to be injured [3]. Transport-related injuries are often minor, but will sometimes be severe enough to warrant euthanasia of the injured horse [4]. Injuries related to transport commonly affect the legs (occurring when the horse contacts the ramp during loading; or against the other leg or part of the trailer/truck during the trip due to loss of balance), the head (due to inadequate height of the trailer or truck) and the tail (occurring when the horse leans its rear against the wall of the truck) [1]. One in four respondents to a survey conducted in South Eastern Australia reported an incident where horses were injured during non-commercial transport and many of these injuries resulted from scrambling and other “flight or fight response” behaviours [5].

Transportation fear seems to be innate in horses [6] triggering “flight or fight response” behaviours and other common signs of activation of the sympathetic system including increased defecation and profuse sweating [7]. An increase in faecal water content and in defecation frequency (diarrhoea) has been previously related to generalize stress responses during travel [8, 9]. Sweating may also be a sign of stress, but when it becomes profuse and associated with hyperthermia, weakness and lethargy, it could indicate heat stroke [10]. Heat stroke can be related to transport events occurring on very hot and humid days. This is not surprising given that in most cases the temperature differences between inside and outside the vehicle usually range from 5.1°C to 9.5°C depending on vehicle speed and the open vent area [11]. Temperature in the vehicle can be close to 10°C warmer than the outside temperature in a stationary vehicle [12]. However, it should be highlighted that fear level can drastically affect the response to transit where a frightened horse may become overheated and sweat, even in comfortable weather conditions [13].

Other diseases related to transport are rhabdomyolysis, laminitis, colic (caused by impaction of large colon, and enterocolitis), and transport pneumonia [1, 14]. Transport pneumonia is one of the most studied transport-related pathologies and in the past two decades many predisposing factors have been identified [15, 16]. For instance, it has been shown that confinement and restraint of the head in an elevated position for 6 and 12 hours causes an accumulation of mucus and bacteria in the lower airways [17].Viral infections transmitted during and after the trip [18] can also contribute to the development of transport pneumonia [19]. Other factors associated with a risk of transport pneumonia include poor internal vehicle environment (e.g. air quality, temperature, humidity) and the lack of appropriate ventilation [20, 21]. The lack of adaptation to transport has also been identified as a contributor to the risk of transport-associated pneumonia. A Japanese study evaluating many stress indicators showed that horses that adapted well to transport were less likely to be affected by pneumonia during extensive transport events (either 36 or 41 hours)[22]. Most of these identified risk factors have been generated from relatively controlled scientific trials, during which horses were managed by scientists and not by members of the equine industry (either owners or transport companies). Thus, there are still many unknown factors in the development of equine transport pneumonia and other transport-related diseases that need to be identified, in particular relating ‘real world’ transportation management strategies and condition to disease risk.

The association between horse management and the development of behavioural and health problems has been often investigated through surveys [3, 23, 24]. Surveys distributed to horse owners/trainers have generally produced excellent response rates (52.5% and 67.2%, respectively) [25, 26], demonstrating a great interest from people associated with horses in research related to the health and wellbeing of their horses. In particular, online surveys have been shown to be an efficient way to gather data from the equine industry (759 in Australia and 4601 in Great Britain return rate) [26, 27]. While transportation is an integral aspect of horse management, to the best of our knowledge a survey has never been conducted across a broad cross section of the Australian equine industry to investigate the associations between transport management practices and health problems.

The aims of this online cross-sectional study were to collect information about transport management and transport-related diseases experienced by members of the equine industry (professional and amateur in all sectors) in Australia and to determine possible associations between them. This study reports these data and comments upon associations that may help those working in the equine industry to instigate changes that will improve the health outcomes of the transported horse.

## Materials and Methods

### Survey

Key design features required to ensure valid questionnaire results, recently reviewed by Dean [28] and Christley [29], were addressed in study design. Detail of the design and distribution of the survey and the description of the demographic characteristics of the study population have been reported previously [4]. Briefly, the survey was digitized using SurveyMonkey (SurveyMonkey Inc., California, USA, www.surveymonkey.com) and it was open from June to September 2015. People involved in any equine industry sector (racing, equestrian and recreational) were invited to take part in the survey if they had organized transport at least monthly over the past two years. An invitation letter and the link to the survey were provided to a wide range of Australian horse sports and organisations, that were asked to publicise the survey. The survey link was also published on equestrian websites and promoted through several horse magazines, relevant Facebook pages and online horse forums. The anonymous survey was organised in 3 sections (requests for a copy of the survey can be made to the corresponding author). The first section contained questions related to respondents’ details (i.e. age, address, equine industry sector, relation with horses, number of horses in care, journey frequency and most common journey duration). The second section focused on their typical management practices related to equine transport (i.e. transport management before, during and after the journey). The last section was on transport-related health problems which the respondents’ horses had experienced in the past two years.

This study focused on the association between respondents’ details, transport management and transport-related health problems. A power calculation (http://statulator.com/samplesize.html) determined that more than 400 survey responses would provide a representative sample of the estimated 400,000 Australians who care for horses [25] with power of 0.8 and α = 0.05 [4]. The survey had a return rate of 987 responses, of which 797 met inclusion criteria (respondent organized transportation at least monthly and provided usable responses to all survey questions). The 797 completed responses were used as dataset for the analysis.

### Outcome variables

The key transport-related health problems of interest were: traumatic injuries (laceration, bruising, swelling); diarrhoea; heat stroke (profuse sweating with hyperthermia and lethargy); muscular problems (tying up), laminitis, transport pneumonia and colic.

### Predictive variables

Predictive variables investigated in this study were: respondent details, and transport management before, during and after the journey (Table 1).

**Table 1:** Predictive variables. Name, description and values of the predictive variables evaluated.

Name	Description	Values
**Respondents details**		
Age	Age of the respondents	20–30, 31–40, 41–50, 51–60, >61
Address	In which Australian state they lived	Australian Capital territory, New South Wales, Northern Territory, Queensland, South Australia, Tasmania, Victoria, Western Australia
Sector	In which sector of the horse industry were involved	Thoroughbred (TB) racing, Standarbred (SB) racing, Equestrian sport, Endurance, Horse breeding, Recreational non-competitive
Background	Relationship with the horses	Professional (involved with horses for financial reward), Amateur (involved with horses as a hobby)
Number of horses	Number of horses present on their propriety in the busiest week of the year	<5, 5–10, 11–30, 31–50, >50 horses
Journey frequency	Frequency of organized transport events	Daily, twice a week, weekly, fortnightly, monthly
Journey duration	The most common journey duration	<2, 2–4, 5–8, >8 hours
**Transport management before journey (BJ)**		
Antibiotics	Administration of antibiotics	Yes, No
Tranquilizers	Administration of tranquilizers	Yes, No
Oral supplements	Administration of electrolytes, vitamins	Yes, No
*Ad libitum* hay/water	*Ad libitum* access to hay and water	Yes, No
Protections	Application of any type of protection equipment (travel boots, bandages)	Yes, No
Rug	Wearing of rug	Yes, No
Health assessment	If and by whom fitness for travel was assessed	A veterinarian, non-veterinary staff, no assessment
Temperature	Assessment of body temperature	Yes, No
Heart rate	Assessment of heart rate	Yes, No
Feeding behaviour	Monitoring of feeding behaviour	Yes, No
Drinking behaviour	Monitoring of drinking behaviour	Yes, No
Weight	Registering body weight	Yes, No
General health	Assessment of general health (e.g. horse coat, demeanour) by visual inspection	Yes, No
**Transport management during journey**		
Vehicle	Vehicle used to transport horses	Two-horse straight trailer, two-horse angle trailer, 3-4-horse angle trailer, gooseneck-trailer, truck
Tying	Horses tied inside the vehicle	Yes, No
Monitoring	If and how horses are monitored	By video camera, at fuel stop, no monitor
Feeding	If horses had access to food and/or water whist travelling	Yes, No
**Transport management after journey (AJ)**		
Health assessment	If and by whom the fitness after travel was assessed	A veterinarian, non-veterinary staff, no assessment
Temperature	Assessment of body temperature	Yes, No
Heart rate	Assessment of heart rate	Yes, No
Feeding behaviour	Monitoring of feeding behaviour	Yes, No
Drinking behaviour	Monitoring of drinking behaviour	Yes, No
Weight	Registering body weight	Yes, No
General health	Assessment of general health (e.g. horse coat, demeanour) by visual inspection	Yes, No
Recovery strategies	If a particular strategy (administration of electrolytes, hand walking) was applied to helping in recovering	Yes, No

### Statistical Analysis

The overall incidence for each transport related problem was calculated by descriptive statistical analysis using GenStat^®^Version 14 (VSN International, Hemel Hempstead, UK). The survey results were analysed by logistic regression using GenStat^®^Version 14 (VSN International, Hemel Hempstead, UK). A model was derived for each transport related problem; the outcome was binary (1/0, affected/non-affected); P values were calculated using Wald Test. Each predictor variable returning a P-value <0.25 from the univariate modelling was considered for inclusion in a multivariate model for that outcome (S1 Table). A step-wise backward elimination procedure was then conducted whereby predictive variables were removed until all variables in the final model had a P-value<0.05 indicating significance. The findings are presented as odds ratio (OR) and confidence interval (95% CI) for each predictive variable value. Wald test’s P values were reported for each association.

## Results

Description of the respondent population, transport management strategies and reported incidence of transport-related problems have been published previously [4]. Briefly, the overall incidence of specific transport related problems is shown in Table 2. Traumatic injuries were the most prevalent transport related problem in this survey, with an incidence of 45% reported. The incidence of transport related diarrhoea was 20%, whilst the incidence of heat stroke, muscular problems, colic and pneumonia associated with transport ranged from 9.2–13.0%. The incidence of transport-related laminitis was <3%.

**Table 2:** Incidence of transport-related health problems.

Transport related problem	Overall incidence (OI)
Traumatic injuries	45.0%
Diarrhoea	20.0%
Muscular problems	13.0%
Heat stroke	10.5%
Colic	10.3%
Transport pneumonia	9.2%
Laminitis	2.9%

Overall incidence for the examined transport-related health problems from the most frequent to the less frequent was traumatic injuries, diarrhoea, muscular problems, heat stroke, colic, transport pneumonia, laminitis.

The results of univariate analyses are provided in (S2–S7 Tables).

The significant results generated from the final multivariate logistic model for the outcome variables are presented in Tables 3–7. The incidence of traumatic injuries related to transport was significantly associated with age of the respondent, maximum number of horses respondents cared for in a given week, administration of tranquilizers, application of any type of protection, the type of vehicle used to transport horses and the general health of the horse after transportation (Table 3).

**Table 3:** Results of the multivariate model with traumatic injuries as the outcome variable. Significant respondents details and transport management risk factors for transport-related traumatic injuries identified using a multivariate logistic regression model (n = 787). Ref = Reference category; s.e. = standard error; OR = odds ratio; CI = 95% confidence interval.

Variable and Category	Estimate	s.e.	OR	95%CI	^a^ P
**Age**					< .001
>61	Ref		1		
51–60	0.17	0.33	1.15	0.60–2.22	
41–50	0.45	0.32	1.57	0.83–2.97	
31–40	0.90	0.33	2.40	1.25–4.61	
20–30	1.52	0.33	4.41	2.31–8.43	
**Number of horses**					< .001
<5	Ref		1		
5–10	0.44	0.20	1.64	1.11–2.43	
11–30	0.31	0.22	1.39	0.89–2.15	
31–50	1.48	0.34	4.23	2.16–8.28	
>51	0.74	0.45	3.69	1.96–6.96	
**Tranquilizers**					0.017
No	Ref		1		
Yes	1.22	0.50	3.30	1.23–8.83	
**Protections**					0.002
No	Ref				
Yes	0.47	0.16	1.65	1.21–2.25	
**Vehicle**					0.028
Truck	Ref		1		
Two horses straight trailer	0.59	0.22	1.82	1.17–2.83	
Two horses angle trailer	-0.01	0.32	0.98	0.52–1.86	
3–4 horses angle trailer	0.347	0.28	1.41	0.81–2.44	
3–4 gooseneck trailer	0.05	0.19	1.76	0.50–2.18	
**General health AJ**					0.004
No	Ref		1		
yes	0.48	0.19	1.76	1.19–2.58	

^**a**^ P value calculated using Wald’s test;AJ: after journey.

**Table 4:** Results of the multivariate model with heat stroke as the outcome variable. Significant respondents details and transport management risk factors for transport related heat stroke identified using a multivariate logistic regression model (n = 787). Ref = Reference category; s.e. = standard error; OR = odds ratio; CI = 95% confidence interval.

Variable and Category	estimate	s.e.	OR	95%CI	^a^ P
**Backgrounds**					
Professionals	Ref				0.045
Amateurs	0.864	0.27	1.75	1.01–3.03	
***Ad libitum* hay/water**					
Yes	Ref				0.002
No	0.864	0.277	2.37	1.37–4.086	

^**a**^ P value calculated using Wald’s test.

**Table 5:** Results of the multivariate model with muscular problem as the outcome variable. Significant respondents details and transport management risk factors for transport related muscular problems identified using a multivariate logistic regression model (n = 787). Ref = Reference category; s.e. = standard error; OR = odds ratio; CI = 95% confidence interval.

Variable and Category	estimate	s.e.	OR	95%CI	^a^ P
**Health assessment BJ**					0.016
A veterinarian	Ref				
Non veterinary staff	0.967	0.734	2.62	0.62–11.09	
No assessment	1.731	0.780	5.64	1.22–26.05	
**Drinking behaviour AJ**					0.031
No	Ref				
Yes	0.557	0.258	1.74	1.05–2.89	

^**a**^ P value calculated using Wald’s test; BJ: before journey; AJ: after journey.

**Table 6:** Results of the multivariate model with laminitis as the outcome variable. Significant respondents details and transport management risk factors for transport related laminitis identified using a multivariate logistic regression model (n = 787). Ref = Reference category; s.e. = standard error; OR = odds ratio; CI = 95% confidence interval.

Variable and Category	estimate	s.e.	OR	95%CI	^a^ P
**Weight BJ**					0.035
No	Ref				
Yes	0.995	0.47	2.70	1.07–6.80	
**Recovery strategies**					0.018
Yes	Ref				
No	1.089	0.46	2.97	1.20–7.33	

^**a**^ P value calculated using Wald’s test; BJ: before journey.

**Table 7:** Results of the multivariate model with transport pneumonia as the outcome variable. Significant respondents details and transport management risk factors for transport pneumonia identified using a multivariate logistic regression model (n = 787). Ref = Reference category; s.e. = standard error; OR = odds ratio; CI = 95% confidence interval.

Variable and Category	estimate	se	OR	95%CI	^a^ P value
**Sector**					0.002
Recreational	Ref				
Endurance	0.80	0.64	2.24	0.63–7.92	
Equestrian Sport	0.41	0.49	1.50	0.57–3.95	
Horse Breeding	0.48	0.60	1.61	0.49–7.92	
SB racing	1.59	0.63	4.91	1.40–17.15	
TB racing	1.84	0.55	6.34	2.12–18.98	
**Number of horse**					0.004
<5	Ref		Ref		
5–10	0.82	0.42	2.27	0.98–5.26	
11–30	0.63	0.45	1.89	0.77–4.63	
31–50	1.22	0.53	3.39	1.18–9.67	
>51	1.90	0.50	6.73	2.50–16.11	
**Journey duration**					< .001
< 2 hours	Ref				
2–4 hours	-0.021	0.34	0.97	0.50–1.90	
4–8 hours	0.92	0.41	2.53	1.13–5.63	
>8 hours	1.84	0.52	6.29	2.26–17.49	
**Temperature AJ**					< .001
No			Ref		
Yes	1.24	0.28	3.46	1.98–6.06	

^**a**^ P value calculated using Wald’s test; SB: Standardbred; TB: Thoroughbred; AJ: after journey.

The incidence of transport related diarrhoea was significantly associated with one predictor variable, that being the background of the respondents. The odds of transport related diarrhoea were 1.5 (CI: 1.01–2.24, P = 0.046) times greater if the carers were amateurs compared to professionals. The incidence of heat stroke related to transportation was significantly associated with the background of the respondents and access to feed and water prior to transport (*ad libitum* hay and water) (Table 4).

Transport-related muscular problems were reported significantly more often if health assessment prior to transport was performed by non-veterinary (lay) persons or not at all, and if drinking behaviour after transportation was monitored (Table 5).

The incidence of laminitis related to transport was significantly greater if body weight was monitored prior to transportation, and was lessened if post transport recovery strategies were used (Table 6)

Four risk factors were significantly associated with the occurrence of transport pneumonia. This condition was reported less commonly by respondents transporting horses for recreational purposes, by respondents caring for fewer than five horses per week, and when journeys were less than 2 hours duration. The condition was reported more commonly when rectal temperature was monitored following transportation (Table 7)

None of the examined predictive variables were significantly associated with the risk of transport related colic; this was true for both univariate and multivariate analyses.

## Discussion

This cross sectional study explored for the first time associations between equine transport management, including information on those caring for the horses, and respondents’ perceptions of transport-related health problems in horses. The occurrence of colic associated with transportation was not associated with any risk factor examined in the current study. Different risk factors were associated with the other transport-related health problems examined. However, the findings of surveys relying upon the retrospective recollection of participants should not be construed as defining causal relationships among factors [24]. The findings of this study add to the knowledge base that can be used in the development of transport guidelines that are designed to improve the welfare of the travelling horse and decrease the risk of disease or injury. Our findings highlight key areas that require empirical further research. It is worth highlighting that the nature of the relationships identified between transport-related health problems and transport management routines in this study cannot be clearly defined [24, 30]. For instance, management practices may have been applied in attempt to identify transport-related problems rather than being the cause. Thus, some of the associations between monitoring strategies and transport-related problems (e.g. visual inspection and injuries; monitoring temperature and transport pneumonia) are more likely a reflection of good practices for prompt identification of these problems, and have been interpreted in this way. Thus, based on the observed associations, these practices might be recommended for identifying animals at risk of diseases and not for preventing transport-related health problems. Individual horse details, sex, breed, age, travel experience have been not considered in this cross sectional study and they should form a critical component of future experimental or observational studies.

This study has a number of limitations common to online surveys [29]. The response rate and power analyses performed can be based only on estimations of the total number of participants in the Australian horse sector [25], as the true number of horse owners cannot be known. Dean [28] has identified sampling bias, non-response bias, recall bias and social acceptability bias as factors that may confound the interpretation of survey data, and all may apply to this study. Although strenuous efforts were made to recruit participants across all components of the Australian horse industry, and numbers of respondents from each sector have been reported, it is not possible to determine whether there is a difference in response rate between sectors (sampling bias). Certainly online survey distribution selected for participants with internet access. Across sectors, respondents who have experienced transport-related problems may have been more motivated to participate (response bias). Arguably, however, the experiences of such a group is best suited to informing discussion of factors associated with injury or disease after transportation. Conversely, potential respondents who are utilising practices that effectively limit the incidence of such problems may not have responded. In the absence of objective data on disease incidence, recall bias may have influenced findings and some observations, particularly the apparent association between respondent age and the number of adverse events might be explained by this factor. As there was no means to document whether a veterinary diagnosis informed participant perceptions or recall, the diagnosis of specific problems associated with transportation must be treated with some circumspection. However, to mitigate this risk, disease categories were deliberately defined in broad terms designed to be meaningful to lay people in the industry. Further, the health problems rating scale is well described and was reduced into binary responses for analysis to limit the effect of individual interpretation. Finally, participants, although anonymous, may have been reluctant to disclose some aspects of their practice in the survey (accountability bias). Given the aim of the study was to survey a large and diverse component of the equine industry to document perceptions about transport management practices and outcomes, this work reports for the first time findings that may be useful not only to safeguarding horse welfare but also to reducing the economic waste related to transport in the equine industry. Our findings add confirmatory evidence to the European [31, 32] and Australian [33] guidelines for equine transportation.

In comparison to horses managed by older respondents, horses managed by respondents younger than 40 years old appeared to be at greater risk of transport related injuries. These findings support the assertions commonly found in the literature that horses should be managed during loading and travelling by experienced and educated people [13, 34, 35].Our finding may be linked not only with limited experience in horse handling but also in driving skills. Driving ability has been identified as a factor associated with transport related injuries, due to erratic driving related to lack of experience impairing the horse’s ability to balance in the trailer [36]. Similarly in cattle, drivers with less than 5 years’ experience reported a higher incidence of injuries associated with transportation [37]. Specialized training in equine transport is not mandatory under the Australian code of transport [33] and, although such training is mandated in Europe [38], to the authors’ knowledge no country has an explicit requirement for a special driving license for live animal transport. Specialised training in how driving styles impact on balance, injuries and stress of the transported animal may be beneficial in reducing transport related injury risk. Traumatic injuries during transportation may involve also horse handlers. An elevated risk of injury has been associated with poor experience, misjudging how to handle a situation, reduced attention caused by distraction, taking a general view, and failing to consider other strategies that may reduce risks [39]. To improve safety for humans and horses, knowledge and experience in horse handling and driving would appear important. The odds of a horse injury associated with transport was 4 times greater for respondents who took care of more than 30 horses. This may be related to the fact that moving larger numbers of horses increases the likelihood of traumatic injuries, supporting the assertion that injuries during transport happen mainly by accident [3].

The higher likelihood of injuries associated with the use of tranquilizers may be related with the fact that tranquilizers can affect the horse’s proprioception and balance, increasing the risk of falling over at loading and during travelling [40]. The results of this study therefore support studies and recommendation that advocate no use of tranquilizers prior to and during journeys [33, 41]. A higher risk of injuries was also associated with the use of protection. Although this finding seems counterintuitive, the use of leg protections and head bumper guards has previously been discouraged during transportation of horses [33, 35]. Protections should be used only on horses completely accustomed to them, they should be properly applied and checked periodically en route, and they should not worn for a long period of time [35]. That is, the use of protective equipment should probably be limited and not universally recommended.

The transport vehicle and its internal design has been previously linked to injuries during animal transportation [42]. Our findings show that the two horse straight trailer design appeared to be associated with elevated risk of transport related injuries compared with truck and gooseneck trailers. This could be related with the reduced stability that trailers have compared with the other vehicles [35]. The elevated risk of injury in straight trailers compared with those in which horses are situated at an angle (~45°) was an interesting finding and reflects recommendations made previously that discourage placing horses so that they are facing in the direction of travel [43, 44]. Horses tend to lose their balance more easily when facing the direction of travel, in particular at abrupt stops. Their posture when facing in this direction also tends to be more rigid and less relaxed, potentially making them more susceptible to injuries [45]. Taking into account that most of the currently used methods and practices for the transportation of horses have been established over a period of time by the demands of the industry, with few governmental or industry standards applicable [35], our findings support the need for a stricter application of standards in horse transport vehicle design [46].

The link in this study between elevated risk of diarrhoea and amateur status of the respondents was an interesting one. Diarrhoea may be a response to an acute stress [7] and equitation science theory suggests that when pressure on the horse is not released correctly, horses tend to be more agitated and show more conflicting behaviours [47]. Thus, horses managed during transportation by less experienced people may be under more stress and consequently experience diarrhoea more frequently. This hypothesis would need to be investigated in more detail through prospective observational studies comparing handling and management procedures of amateurs and professionals on such potential physiological stress responses, as diarrhoea. Horses transported by amateur respondents were also more likely to experience transport-related heat stroke in their horses. This may have also been due to greater emotional stress when horses were not managed correctly. Agitated horses have been reported to suffer heat stroke even when weather conditions are said to be comfortable [13].

The risk of transport related heat stroke was elevated when water and hay were restricted before transport. Dehydration can impair equine thermoregulation and lead to heat stroke [10]. Offering *ad libitum* water and hay prior to the journey, can act to insure good electrolyte balance and hydration status, enabling horses to better handle environmental conditions and stresses that could result in significant dehydration and electrolyte losses during transportation. Due to Australia’s climate, horses can at times be transported in extreme heat and high humidity. Therefore ensuring horses are properly hydrated seems a ‘common sense’ strategy to enhance their wellbeing during travel. Therefore, hay and water restrictions prior to transport should be avoided. This finding complements recent published guidelines on watering during transportation of *equidae* [32].

An elevated risk of muscular problems associated with transport was observed when pre transport health checks were lacking. Electromyography studies have demonstrated that the muscles of the horse are in continuous activity during transport as they adjust posture and balance [36]. Elevations in muscle enzymes, such as creatine kinase (CK), have been noted even after trips of relatively modest duration (3 hours) [48], whilst transportation events spanning 300km produce muscle stress equivalent to a 1,500 m canter [49]. The evaluation of the fitness for travel is mandatory and simple guidelines have been recently published by the world organisation for animal health (OIE) [31]. We therefore recommend that fitness for travel be assessed using these guidelines for all horses before every trip, with assessment by a qualified veterinarian if there is any doubt about an individual horse [31] or before journeys longer than 20 hours [14].

Laminitis is a pathology which can lead to death or euthanasia of the horse, and it has been associated with a number of factors such as endotoxemia and severe dehydration after colic, placental retention and transport [10]. In this study, the risk of laminitis associated with transport was significantly elevated when there was a lack of a post transport recovery strategy, such as rehydration and walking. There is a lack of research testing the effects of specific recovery strategies on the risk of transport-related diseases, however rehydration, hand walking and housing in paddocks have been recommended [1, 8, 41]. Transport pneumonia was associated with the respondents’ sector, with racing horses at higher risk than pleasure horses. This could be explained in some way by bred predisposition to transport pneumonia, as reported for the Thoroughbred [16], but also may be related to the combined effects of strenuous exercise and transportation, which are both seen as predisposition factors for equine pneumonia [15]. Journey duration has been previously associated with increased risk of developing transport pneumonia [14], as was reported in the current study. The association between number of horses in care and pneumonia could be interpreted in two ways. The first is just the likelihood of disease increased proportionally with the number of horses, such as for injuries. The second is the typology of horse stable. Horse stables with more horses might have more cases of transport pneumonia because they tend to travel on longer distance or could more easily transmit pathogens among horses. Interestingly, this study showed an association between pneumonia and the monitoring of temperature after the journey. This result may be related to the use of this tool in the early detection of disease, with an elevated temperature a well-recognized early indicator of transport related respiratory disease [50]. The practice of monitoring the body temperature of horses after transport is commonly recommended within the industry [8, 20], and these results should not discourage this practice but as an evidence in the utility of applying it.

Even though transport-related colic was reported by more than 10% of the respondents, no associations were found with the variables investigated in this study. Colic is a common problem in horses and it may be caused by a variety of factors (e.g. dehydration, abrupt change in diet, ingestion of sand while grazing). Transport has been identified as a risk factor for simple colonic obstruction and distension colic [51] and for salmonellosis [52], but previous colic, age (2–10 years), increased concentrate intake, change in feeding and medical treatment have been identified as major risk factors in the development of colic [53]. We can therefore speculate that transportation may worsen gastrointestinal conditions and contribute to colic development, in particular in association with poor watering [32] and inappropriate diet management [54]. However, more research is required to identify other unknown risk factors in transport-related colic.

## Conclusions

This study identified various potential factors contributing to a number of key transport-related health problems in horses. Age, experience and professionalism seemed to influence the risk of injury and other more directly stress related outcomes, such as diarrhoea and heat stroke. Among the various strategies investigated to prepare the horse for transport, offering *ad libitum* water and hay and an appropriate health check prior to transportation seemed to reduce the risk of heat stroke and muscular problems. The monitoring of the horses after transport, in particular observing their behaviour and recording their body temperature, appeared to increase the risk of disease but such outcomes should not be interpreted as causal but rather are likely to reflect early diagnosis and intervention that would likely benefit the horse’s ability to recover from transport related problem. Our findings add confirmatory evidence on the importance of best practices during transportation to reduce the negative impacts of transportation on horse health and welfare, confirming previously suggested guidelines and suggesting also some new practices. However, since it is obvious that transport related problems are multifactorial and this study is only a survey, our findings need to be interpreted with caution. The factors identified in this study warrant future experimental exploration in order to test the efficacy of some strategies and generate more concrete guidelines to safeguard the transported equine.

## Supporting Information

S1 Table Wald test’s P values generated from univariate regression analysis. (DOCX)

S2 Table Results of the univariate regression analysis with injuries as the outcome. Respondents’ details and transport management risk factors for transport related injuries with a Wald test P value less than 0.250 identified using univariate logistic regression. In the third and fourth column the frequency of the respondent (not reporting and reporting injuries) are reported as total number (n) and percentage in each category. Odds ratio (OR); 95% confidence interval (95%CI); ^a^ P value calculated using Wald’s test (P).
(DOCX)

S3 Table Results of the univariate regression analysis with diarrhoea as the outcome. Respondents’ details and transport management risk factors for transport related diarrhea with a Wald test P value less than 0.250 identified using univariate logistic regression. In the third and fourth column the frequency of the respondent (not reporting and reporting diarrhea) are reported as total number (n) and percentage in each category. Odds ratio (OR); 95% confidence interval (95%CI); ^a^ P value calculated using Wald’s test (P).
(DOCX)

S4 Table Results of the univariate regression analysis with muscular problem as the outcome. Respondents’ details and transport management risk factors for transport related muscular problems with a Wald test P value less than 0.250 identified using univariate logistic regression. In the third and fourth column the frequency of the respondent (not reporting and reporting muscular problems) are reported as total number (n) and percentage in each category. Odds ratio (OR); 95% confidence interval (95%CI); ^a^ P value calculated using Wald’s test (P).
(DOCX)

S5 Table Results of the univariate regression analysis with heat stroke as the outcome. Respondents’ details and transport management risk factors for transport related heat stroke with a Wald test P value less than 0.250 identified using univariate logistic regression. In the third and fourth column the frequency of the respondent (not reporting and reporting heat stroke) are reported as total number (n) and percentage in each category. Odds ratio (OR); 95% confidence interval (95%CI); ^a^ P value calculated using Wald’s test (P).
(DOCX)

S6 Table Results of the univariate regression analysis with laminitis as the outcome. Respondents’ details and transport management risk factors for transport related laminitis with a Wald test P value less than 0.250 identified using univariate logistic regression. In the third and fourth column the frequency of the respondent (not reporting and reporting laminitis) are reported as total number (n) and percentage in each category. Odds ratio (OR); 95% confidence interval (95%CI); ^a^ P value calculated using Wald’s test (P).
(DOCX)

S7 Table Results of the univariate regression analysis with transport pneumonia as the outcome. Respondents’ details and transport management risk factors for transport pneumonia with a Wald test P value less than 0.250 identified using univariate logistic regression. In the third and fourth column the frequency of the respondent (not reporting and reporting pneumonia) are reported as total number (n) and percentage in each category. Odds ratio (OR); 95% confidence interval (95%CI); ^a^ P value calculated using Wald’s test (P).
(DOCX)

S1 Data Raw data of the survey. (CSV)
