# Supplementary material for: A Survey on Transport Management Practices Associated with Injuries and Health Problems in Horses
Source: PLoS One. 2016 Sep 2;11(9):e0162371. doi: 10.1371/journal.pone.0162371 (PMC5010189; doi:10.1371/journal.pone.0162371)
Supplement: S1 Table — (DOCX) [file pone.0162371.s001.docx]

**S1 Table**. **Wald test’s P values generated from univariate regression analysis.**

| **Predictive**  **Variables** | **Traumatic**  **Injuries** | **Diarrhoea** | **Muscular Problem** | **Heat stroke** | **Laminitis** | **Transport**  **Pneumonia** | **Colic** |
| --- | --- | --- | --- | --- | --- | --- | --- |
| **Age** | <0.001 | 0.224 | 0.932 | 0.378 | 0.387 | 0.190 | 0.331 |
| **Address** | 0.159 | 0.176 | 0.090 | 0.591 | 0.562 | 0.669 | 0.808 |
| **Sector** | 0.120 | 0.246 | 0.534 | 0.879 | 0.164 | <0.001 | 0.588 |
| **Background** | 0.144 | 0.046 | 0.430 | 0.063 | 0.630 | <0.001 | 0.760 |
| **Number of horses** | <0.001 | 0.756 | 0.690 | 0.167 | 0.412 | <0.001 | 0.171 |
| **Journey frequency** | 0.392 | 0.796 | 0.381 | 0.769 | 0.207 | <0.001 | 0.317 |
| **Journey duration** | 0.593 | 0.846 | 0.423 | 0.696 | 0.882 | <0.001 | 0.625 |
| **Antibiotics** | 0.099 | 0.994 | 0.409 | 0.429 | 0.655 | 0.155 | 0.693 |
| **Tranquilizers** | 0.009 | 0.317 | 0.198 | 0.358 | 0.737 | 0.838 | 0.701 |
| **Oral supplements** | 0.535 | 0.906 | 0.601 | 0.864 | 0.278 | <0.001 | 0.780 |
| ***Ad libitum* hay/water** | 0.237 | 0.152 | 0.074 | 0.003 | 0.135 | 0.421 | 0.316 |
| **Protections** | <0.001 | 0.356 | 0.847 | 0.930 | 0.454 | 0.325 | 0.773 |
| **Wearing of rug** | 0.166 | 0.886 | 0.800 | 0.892 | 0.781 | 0.004 | 0.968 |
| **Health assessment BJ** | 0.482 | 0.753 | 0.085 | 0.745 | 0.786 | 0.030 | 0.274 |
| **Temperature BJ** | 0.039 | 0.753 | 0.630 | 0.316 | 0.331 | <0.001 | 0.937 |
| **Heart rate BJ** | 0.404 | 0.530 | 0.799 | 0.413 | 0.912 | 0.449 | 0.459 |
| **Feeding behaviour BJ** | 0.079 | 0.633 | 0.344 | 0.458 | 0.725 | 0.003 | 0.373 |
| **Drinking behaviour BJ** | 0.596 | 0.583 | 0.842 | 0.990 | 0.522 | 0.002 | 0.345 |
| **Weight BJ** | 0.055 | 0.178 | 0.134 | 0.354 | 0.080 | 0.550 | 0.401 |
| **General health BJ** | 0.007 | 0.744 | 0.781 | 0.646 | 0.339 | 0.720 | 0.825 |
| **Vehicle** | 0.064 | 0.929 | 0.829 | 0.697 | 0.773 | <0.001 | 0.462 |
| **Tying** | 0.452 | 0.677 | 0.983 | 0.097 | 0.516 | 0.644 | 0.395 |
| **Monitoring** | 0.128 | 0.167 | 0.201 | 0.418 | 0.586 | 0.95 | 0.151 |
| **Feeding** | 0.752 | 0.813 | 0.052 | 0.868 | 0.093 | 0.025 | 0.788 |
| **Health assessment AJ** | 0.112 | 0.365 | 0.243 | 0.645 | 0.846 | 0.095 | 0.713 |
| **Temperature AJ** | 0.016 | 0.540 | 0.308 | 0.065 | 0.346 | <0.001 | 0.353 |
| **Heart rate AJ** | 0.613 | 0.315 | 0.680 | 0.892 | 0.915 | 0.493 | 0.656 |
| **Feeding behaviour AJ** | 0.004 | 0.185 | 0.401 | 0.559 | 0.781 | 0.004 | 0.968 |
| **Drinking behaviour AJ** | 0.004 | 0.104 | 0.194 | 0.967 | 0.941 | 0.003 | 0.977 |
| **Weight AJ** | 0.006 | 0.874 | 0.395 | 0.602 | 0.233 | 0.777 | 0.167 |
| **General health AJ** | <0.001 | 0.224 | 0.461 | 0.187 | 0.347 | 0.427 | 0.713 |
| **Recovery Strategies** | 0.006 | 0.248 | 0.821 | 0.707 | 0.034 | 0.022 | 0.759 |

Wald test’s P values calculated in the univariate regression analyses for all predictive variables on the following outcomes: traumatic injuries, colic, diarrhoea, heat stroke, laminitis, transport pneumonia (BJ: before journey; AJ: after journey).
