# Supplementary material for: A Survey on Transport Management Practices Associated with Injuries and Health Problems in Horses
Source: PLoS One. 2016 Sep 2;11(9):e0162371. doi: 10.1371/journal.pone.0162371 (PMC5010189; doi:10.1371/journal.pone.0162371)
Supplement: S2 Table — Respondents’ details and transport management risk factors for transport related injuries with a Wald test P value less than 0.250 identified using univariate logistic regression. In the third and fourth column the frequency of the respondent (not reporting and reporting injuries) are reported as total number (n) and percentage in each category. Odds ratio (OR); 95% confidence interval (95%CI); a P value calculated using Wald’s test (P). (DOCX) [file pone.0162371.s002.docx]

**S2 Table. Results of the univariate regression analysis with injuries as the outcome**.

| **Variable** | **Category** | **No Injuries**  **n(%)** | **Injuries n(%)** | **OR** | **95%CI** | **P ^a^** |
| --- | --- | --- | --- | --- | --- | --- |
| Age | >61 | 47(71.2) | 19(28.8) | Ref | 1 | <.001 |
|  | 51-60 | 115(68.9) | 52(31.1) | 1.11 | 0.59-2.08 |  |
|  | 41-50 | 122(61.6) | 76(38.4) | 1.54 | 0.84-2.81 |  |
|  | 31-40 | 79(49.4) | 81(50.6) | 2.53 | 1.37-4.69 |  |
|  | 20-30 | 67(35.0) | 124(65) | 4.57 | 2.49-8.40 |  |
| Address | ACT | 17 (62.9) | 10 (37.1) | Ref | 1 | 0.159 |
|  | NSW | 180 (56.6) | 138(43.4) | 1.30 | 0.57-2.93 |  |
|  | NT | 18 (72) | 7(28) | 0.66 | 0.20-2.12 |  |
|  | QLD | 43(45.3) | 52(54.7) | 2.05 | 0.85-4.94 |  |
|  | SA | 26(48.1) | 28(51.9) | 1.83 | 0.71-4.71 |  |
|  | TAS | 13(72.2) | 5(27.8) | 0.65 | 0.17-2.37 |  |
|  | VIC | 101(54.3) | 85(45.7) | 1.43 | 0.62-3.28 |  |
|  | WA | 40(21.5) | 34(78.5) | 1.44 | 0.58-3.56 |  |
| Sector | Recreational | 104 (54.1) | 88(45.9) | Ref | 1 | 0.120 |
|  | Endurance | 34(66.7) | 17(33.3) | 0.59 | 0.30-1.12 |  |
|  | Equestrian Sport | 218(56.0) | 171(44.0) | 0.92 | 0.65-1.31 |  |
|  | Horse Breeding | 34(46.6) | 39(53.4) | 1.35 | 0.78-2.32 |  |
|  | SB racing | 22(64.7) | 12(35.3) | 0.64 | 0.30-1.37 |  |
|  | TB racing | 26(44.8) | 32(55.2) | 1.45 | 0.80-2.62 |  |
| Backgrounds | Professionals | 123(51.0) | 118(49.0) | Ref | 1 | 0.144 |
|  | Amateurs | 315(56.6) | 241(43.4) | 0.79 | 0.58-1.08 |  |
| Horse Number | <5 | 207(60.7) | 134(39.3) | Ref | 1 | <.001 |
|  | 5-10 | 102(53.1) | 90(46.9) | 1.36 | 0.95-1.94 |  |
|  | 11-30 | 85(57.8) | 62(42.2) | 1.12 | 0.76-1.66 |  |
|  | 31-50 | 20(36.4) | 35(63.6) | 2.70 | 1.50-4.87 |  |
|  | >51 | 24(38.7) | 38(61.3) | 2.44 | 1.40-4.25 |  |
| Antibiotics | No | 433(53.4) | 349(44.6) | Ref | 1 | 0.099 |
|  | Yes | 5(33.3) | 10(66.7) | 2.48 | 0.84-7.30 |  |
| Tranquilizers | No | 431(54.0) | 341(42.7) |  |  | 0.009 |
|  | Yes | 7(0.8) | 18(2.2) | 3.25 | 1.34-7.85 |  |
| Protections | No | 278(34.8) | 181(22.7) | Ref | 1 | <.001 |
|  | Yes | 160(20.0) | 178(22.3) | 1.70 | 1.28-2.62 |  |
| Rugs | No | 288(56.8) | 219(43.2) | Ref | 1 |  |
|  | Yes | 150(51.7) | 140(48.3) | 1.22 | 0.91-1.64 |  |
| Temperature BJ | No | 337(42.2) | 253(31.7) |  |  | 0.039 |
|  | Yes | 101(12.6) | 106(13.2) | 1.39 | 1.01-1.92 |  |
| Feeding Behavior BJ | No | 223(55.9) | 176(44.1) | Ref | 1 | 0.079 |
|  | Yes | 215(54.0) | 183(46.0) | 1.28 | 0.97-1.69 |  |
| Weight BJ | No | 375(85.6) | 63(14.4) | Ref | 1 | 0.055 |
|  | Yes | 289(80.5) | 70(19.5) | 1.44 | 0.99-2.09 |  |
| General health BJ | No | 113(14.1) | 64(8.0) |  |  | 0.007 |
|  | Yes | 325(40.7) | 295(37.0) | 1.60 | 1.13-2.26 |  |
| Vehicle | Truck | 96(60.0) | 64(40.0) | Ref | 1 | 0.064 |
|  | Two horses straight trailer | 207(50.9) | 199(49.1) | 1.44 | 0.99-2.09 |  |
|  | Two horses angle trailer | 48(64.0) | 27(36.0) | 0.84 | 0.47-1.48 |  |
|  | 3-4 horses angle trailer | 56(51.8) | 52(48.2) | 1.39 | 0.85-2.27 |  |
|  | 3-4 gooseneck trailer | 31(64.5) | 17(35.5) | 0.82 | 0.42-1.60 |  |
| Monitoring | No monitor | 176(60.7) | 114(39.3) | Ref | 1 | 0.128 |
|  | By camera | 81(51.9) | 75(48.1) | 1.42 | 0.96-2.11 |  |
|  | At fuel stop | 151(53.9) | 129(46.1) | 1.31 | 0.94-1.84 |  |
| Health assessment AJ | A veterinarian | 24(53.3) | 21(46.7) | Ref | 1 | 0.112 |
|  | Non veterinary staff | 354(53.6) | 306(46.4) | 0.98 | 0.53-1.81 |  |
|  | No assessment | 60(65.2) | 32(34.8) | 0.60 | 0.29-1.25 |  |
| Temperature AJ | No | 338(42.4) | 250(31.6) |  |  | 0.016 |
|  | Yes | 100(12.5) | 109(13.6) | 1.47 | 1.07-2.02 |  |
| Feeding Behavior AJ | No | 179(22.4) | 111(13.9) |  |  | 0.004 |
|  | yes | 259(32.4) | 248(31.1) | 1.54 | 1.15-2.07 |  |
| Drinking Behavior AJ | No | 175(21.9) | 108(13.5) |  |  | 0.004 |
|  | yes | 263(32.9) | 251(31.4) | 1.54 | 1.15-2.07 |  |
| Weight AJ | No | 364(58.2) | 270(33.8) |  |  | 0.006 |
|  | Yes | 74(9.2) | 89(11.1) | 1.62 | 1.14-2.29 |  |
| General Health AJ | No | 120(67.4) | 58(32.6) | Ref | 1 | <.001 |
|  | Yes | 318(51.4) | 301(48.6) | 1.95 | 1.38-2.77 |  |
| Recovery  Strategies | No | 226(28.3) | 150(18.8) |  |  | 0.006 |
|  | Yes | 212(26.5) | 209(26.2) | 1.48 | 1.21-1.96 |  |

Respondents’ details and transport management risk factors for transport related injuries with a Wald test P value less than 0.250 identified using univariate logistic regression. In the third and fourth column the frequency of the respondent (not reporting and reporting injuries) are reported as total number (n) and percentage in each category. Odds ratio (OR); 95% confidence interval (95%CI); ^a^ P value calculated using Wald’s test (P). ACT: Australian Capital Territory; NSW: New South Wales; NT: Northern Territory; QLD: Queensland; SA: South Australia; TAS: Tasmania, VIC: Victoria; WA: Western Australia; SB: Standardbred, TB: Thoroughbred; BJ: before journey; AJ: after journey.
