# Supplementary material for: A Survey on Transport Management Practices Associated with Injuries and Health Problems in Horses
Source: PLoS One. 2016 Sep 2;11(9):e0162371. doi: 10.1371/journal.pone.0162371 (PMC5010189; doi:10.1371/journal.pone.0162371)
Supplement: S4 Table — Respondents’ details and transport management risk factors for transport related muscular problems with a Wald test P value less than 0.250 identified using univariate logistic regression. In the third and fourth column the frequency of the respondent (not reporting and reporting muscular problems) are reported as total number (n) and percentage in each category. Odds ratio (OR); 95% confidence interval (95%CI); a P value calculated using Wald’s test (P). (DOCX) [file pone.0162371.s004.docx]

**S4 Table. Results of the univariate regression analysis with muscular problem as the outcome.**

| **Variable** | **Category** | **No Muscular problems n(%)** | **Muscular**  **Problems**  **n(%)** | **OR** | **95%CI** | **^a^ P** |
| --- | --- | --- | --- | --- | --- | --- |
| Address | ACT | 25(92.6) | 2(7.3) | Ref | 1 | 0.090 |
|  | NSW | 282(88.7) | 36(11.3) | 1.59 | 0.36-7.01 |  |
|  | NT | 21(84.0) | 4(16.0) | 2.38 | 0.39-14.31 |  |
|  | QLD | 75(78.9) | 20(21.1) | 3.33 | 0.72-15.27 |  |
|  | SA | 48(88.9) | 6(11.1) | 1.56 | 0.29-8.31 |  |
|  | TAS | 14(77.7) | 4(22.3) | 3.57 | 0.58-21.98 |  |
|  | VIC | 168(90.3) | 18(9.7) | 1.33 | 0.29-6.12 |  |
|  | WA | 60(81.1) | 14(18.9) | 2.91 | 0.61-13.78 |  |
| *Ad libitum* hay/water | Yes | 263(89.8) | 30(10.2) | Ref | 1 | 0.074 |
|  | No | 430(85.3) | 74(14.7) | 1.50 | 0.96-2.36 |  |
| Health assessment BJ | A veterinarian | 36(94.7) | 2(5.3) | Ref | 1 | 0.085 |
|  | Non veterinary staff | 570(87.4) | 82(12.6) | 2.58 | 0.61-10.92 |  |
|  | No assessment | 87(81.3) | 20(18.7) | 4.13 | 0.92-18.56 |  |
| Weight BJ | No | 572(86.1) | 92(13.9) | Ref | 1 | 0.134 |
|  | Yes | 121(90.9) | 12(9.1) | 0.61 | 0.32-1.16 |  |
| Monitoring | No monitor | 244(84.1) | 46(15.9) | Ref | 1 | 0.201 |
|  | By camera | 140(89.7) | 16(10.3) | 0.60 | 0.33-1.11 |  |
|  | At fuel stop | 246(87.9) | 34(12.1) | 0.73 | 0.45-1.18 |  |
| Feeding | No | 258(84.0) | 49(16.0) | Ref | 1 | 0.052 |
|  | Yes | 386(88.9) | 48(11.1) | 0.65 | 0.42-1.00 |  |
| Health assessment AJ | A veterinarian | 43(95.5) | 2(4.5) | Ref | 1 | 0.243 |
|  | Non veterinary staff | 570(86.4) | 90(13.6) | 3.39 | 0.81-14.12 |  |
|  | No assessment | 80(86.9) | 12(13.1) | 3.22 | 0.69-14.94 |  |
| Drinking behavior AJ | No | 252(89.0) | 31(11.0) | Ref | 1 | 0.194 |
|  | Yes | 441(85.8) | 73(14.2) | 1.34 | 0.86-2.10 |  |

Respondents’ details and transport management risk factors for transport related muscular problems with a Wald test P value less than 0.250 identified using univariate logistic regression. In the third and fourth column the frequency of the respondent (not reporting and reporting muscular problems) are reported as total number (n) and percentage in each category. Odds ratio (OR); 95% confidence interval (95%CI); ^a^ P value calculated using Wald’s test (P). ACT: Australian Capital Territory; NSW: New South Wales; NT: Northern Territory; QLD Queensland; SA: South Australia; TAS: Tasmania, VIC: Victoria; WA: Western Australia; BJ: before journey; AJ: after journey.
