# Supplementary material for: A Survey on Transport Management Practices Associated with Injuries and Health Problems in Horses
Source: PLoS One. 2016 Sep 2;11(9):e0162371. doi: 10.1371/journal.pone.0162371 (PMC5010189; doi:10.1371/journal.pone.0162371)
Supplement: S6 Table — Respondents’ details and transport management risk factors for transport related laminitis with a Wald test P value less than 0.250 identified using univariate logistic regression. In the third and fourth column the frequency of the respondent (not reporting and reporting laminitis) are reported as total number (n) and percentage in each category. Odds ratio (OR); 95% confidence interval (95%CI); a P value calculated using Wald’s test (P). (DOCX) [file pone.0162371.s006.docx]

**S6 Table. Results of the univariate regression analysis with laminitis as the outcome.**

| **Variable** | **Category** | **No**  **Laminitis** | **Laminitis** | **OR** | **95%CI** | **^a^ P** |
| --- | --- | --- | --- | --- | --- | --- |
| Sector | Recreational | 184(95.8) | 8(4.2) | Ref | 1 | 0.164 |
|  | Endurance | 50(98.0) | 1(2.0) | 0.46 | 0.05-3.71 |  |
|  | Equestrian Sport | 382(98.2) | 7(1.8) | 0.42 | 0.15-1.17 |  |
|  | Horse Breeding | 72(98.6) | 1(1.4) | 0.31 | 0.04-2.49 |  |
|  | SB racing | 31(91.2) | 3(8.8) | 2.22 | 0.55-8.85 |  |
|  | TB racing | 55(94.8) | 3(5.2) | 1.25 | 0.32-4.89 |  |
| Journey frequency | Monthly | 248(96.5) | 9(3.5) | Ref | 1 | 0.207 |
|  | Fortnightly | 160(99.4) | 1(0.6) | 0.17 | 0.22-1.33 |  |
|  | Weekly | 194(97.0) | 6(3.0) | 0.85 | 0.29-2.43 |  |
|  | Twice a week | 121(97.6) | 3(2.4) | 0.68 | 0.18-2.56 |  |
|  | Daily | 51(92.7) | 4(7.3) | 2.16 | 0.64-7.28 |  |
| *Ad libitum* hay/water | Yes | 288(98.3) | 5(1.7) | Ref | 1 | 0.135 |
|  | No | 486(96.4) | 18(3.6) | 2.13 | 0.79-5.75 |  |
| Weight BJ | No | 618(97.5) | 16(3.5) | Ref | 1 | 0.080 |
|  | Yes | 156(95.7) | 7(4.3) | 2.25 | 0.90-5.57 |  |
| Feeding | No | 295(96.1) | 12(3.9) | Ref | 1 | 0.093 |
|  | Yes | 426(98.2) | 8(1.8) | 0.46 | 0.18-1.13 |  |
| Weight AJ | No | 618(97.5) | 16(2.5) | Ref | 1 | 0.233 |
|  | Yes | 156(95.7) | 7(4.3) | 1.73 | 0.70-4.28 |  |
| Recovery  strategies | Yes | 414(98.3) | 7(1.7) | Ref | 1 | 0.003 |
|  | No | 360(95.7) | 16(4.3) | 2.62 | 1.07-6.4 |  |

Respondents’ details and transport management risk factors for transport related laminitis with a Wald test P value less than 0.250 identified using univariate logistic regression. In the third and fourth column the frequency of the respondent (not reporting and reporting laminitis) are reported as total number (n) and percentage in each category. Odds ratio (OR); 95% confidence interval (95%CI); ^a^ P value calculated using Wald’s test (P); SB: Standardbred, TB: Thoroughbred; BJ: before journey; AJ: after journey.
