# Supplementary material for: A Survey on Transport Management Practices Associated with Injuries and Health Problems in Horses
Source: PLoS One. 2016 Sep 2;11(9):e0162371. doi: 10.1371/journal.pone.0162371 (PMC5010189; doi:10.1371/journal.pone.0162371)
Supplement: S7 Table — Respondents’ details and transport management risk factors for transport pneumonia with a Wald test P value less than 0.250 identified using univariate logistic regression. In the third and fourth column the frequency of the respondent (not reporting and reporting pneumonia) are reported as total number (n) and percentage in each category. Odds ratio (OR); 95% confidence interval (95%CI); a P value calculated using Wald’s test (P). (DOCX) [file pone.0162371.s007.docx]

**S7 Table. Results of the univariate regression analysis with transport pneumonia as the outcome.**

| **Variable** | **Category** | **No Transport**  **Pneumonia** | **Transport**  **Pneumonia** | **OR** | **95%CI** | ^a^ **P** |
| --- | --- | --- | --- | --- | --- | --- |
| Age | >61 | 59(89.4) | 7(10.6) | Ref | 1 | 0.190 |
|  | 51-60 | 150(89.2) | 17(10.8) | 0.95 | 0.37-2.42 |  |
|  | 41-50 | 175(88.4) | 23(11.6) | 1.10 | 0.45-2.71 |  |
|  | 31-40 | 145(90.6) | 15(9.4) | 0.87 | 0.33-2.24 |  |
|  | 20-30 | 182(95.3) | 9(4.7) | 0.41 | 0.14-1.16 |  |
| Sector | Recreational | 186(96.9) | 6(3.1) | Ref | 1 | <.001 |
|  | Endurance | 45(88.2) | 6(1.8) | 4.12 | 1.29-13.20 |  |
|  | Equestrian Sport | 369(94.8) | 20(5.2) | 1.67 | 0.67-4.16 |  |
|  | Horse Breeding | 61(83.6) | 12(6.4) | 6.08 | 2.23-16.62 |  |
|  | SB racing | 27(79.4) | 7(20.6) | 8.02 | 2.55-25.26 |  |
|  | TB racing | 36(62.1) | 22(37.9) | 18.92 | 7.29-49.03 |  |
| Backgrounds | Amateur | 553(96.0) | 23(4.0) | Ref | 1 | <.001 |
|  | Professionals | 191(79.2) | 50(20.8) | 6.06 | 3.61-10.17 |  |
| Horse Number | <5 | 330(96.7) | 11(3.3) | Ref | 1 | <.001 |
|  | 5-10 | 177(92.2) | 15(7.8) | 2.53 | 1.15-5.58 |  |
|  | 11-30 | 132(89.8) | 15(10.2) | 3.40 | 1.54-7.52 |  |
|  | 31-50 | 45(81.8) | 10(18.2) | 6.65 | 2.70-16.40 |  |
|  | >51 | 40(64.5) | 22(35.5) | 16.48 | 7.53-36.07 |  |
| Journey  frequency | Monthly | 238(92.6) | 19(7.4) | Ref | 1 | <.001 |
|  | Fortnightly | 155(96.3) | 6(3.7) | 0.48 | 0.19-1.22 |  |
|  | Twice a week | 108(87.1) | 16(12.9) | 1.85 | 0.91-3.74 |  |
|  | Weekly | 186(93) | 14(7) | 0.94 | 0.46-1.9 |  |
|  | Daily | 37(67.3) | 18(32.7) | 6.09 | 2.9-12.65 |  |
| Journey  duration | < 2hours | 434(93.7) | 29(6.3) | Ref | 1 | <.001 |
|  | 2-4 hours | 205(90.3) | 22(9.7) | 1.60 | 0.90-2.86 |  |
|  | 5-8 hours | 65(82.3) | 14(17.7) | 3.22 | 1.61-6.41 |  |
|  | > 8 hours | 20(71.4) | 8(28.6) | 5.98 | 2.43-14.72 |  |
| Antibiotics | No | 712(91.0) | 70(9.0) | Ref | 1 | 0.155 |
|  | Yes | 12(80.0) | 3(20.0) | 2.54 | 0.70-9.20 |  |
| Oral supplements | No | 613(92.9) | 47(7.1) | Ref | 1 | <.001 |
|  | Yes | 111(81.0) | 26(19.0) | 3.05 | 1.18-5.13 |  |
| Wearing rugs | No | 449(88.6) | 58(11.4) | Ref |  | 0.004 |
|  | Yes | 275(94.8) | 15(5.2) | 2.36 | 1.31-4.25 |  |
| Health assessment BJ | No assessment | 104(97.2) | 3(2.8) | Ref |  | 0.030 |
|  | Non veterinary staff | 588(90.2) | 64(9.8) | 6.48 | 1.5-26.50 |  |
|  | A veterinarian | 32(84.2) | 6(15.8) | 3.76 | 1.20-11.71 |  |
| Temperature  BJ | No | 562(95.2) | 28(4.8) | Ref | 1 | <.001 |
|  | Yes | 162(78.3) | 45(21.7) | 5.57 | 3.37-9.20 |  |
| Feeding Behaviour BJ | No | 364(94.0) | 23(6.0) | Ref | 1 | 0.003 |
|  | yes | 360(87.8) | 50(12.2) | 2.19 | 1.31-3.67 |  |
| Drinking Behaviour BJ | No | 375(93.9) | 24(6.1) | Ref | 1 | 0.002 |
|  | yes | 349(87.7) | 49(12.3) | 2.19 | 1.31-3.65 |  |
| Vehicle | Two horses angle trailer | 71(94.7) | 4(5.3) | Ref | 1 | <.001 |
|  | Truck | 127(79.4) | 33(20.6) | 4.61 | 1.57-13.51 |  |
|  | Two horses straight trailer | 388(95.6) | 18(4.4) | 0.82 | 0.27-2.49 |  |
|  | 3-4 horses angle trailer | 95(87.9) | 13(12.1) | 2.42 | 0.76-7.74 |  |
|  | 3-4 gooseneck trailer | 43(89.6) | 5(10.4) | 2.06 | 0.52-8.09 |  |
| Do you offer food | Yes | 404(93.1) | 30(6.9) | Ref | 1 | 0.025 |
|  | No | 271(88.3) | 36(11.7) | 1.78 | 1.07-2.97 |  |
| Health assessment AJ | A veterinarian | 39(86.7) | 6(13.3) | Ref | 1 | 0.095 |
|  | Non veterinary staff | 596(90.3) | 64(9.7) | 0.69 | 0.28-1.71 |  |
|  | No assessment | 89(96.7) | 3(3.3) | 0.21 | 0.05-0.90 |  |
| Temperature AJ | No | 559(95.1) | 29(4.9) | Ref | 1 | <.001 |
|  | Yes | 165(78.9) | 44(21.1) | 5.14 | 3.12-8.4 |  |
| Feeding Behaviour AJ | No | 275(94.8) | 15(5.2) | Ref | 1 | 0.004 |
|  | yes | 449(88.6) | 58(11.4) | 2.36 | 1.31-4.25 |  |
| Drinking Behaviour AJ | No | 269(95.1) | 14(4.9) | Ref | 1 | 0.003 |
|  | yes | 455(88.5) | 59(11.5) | 2.49 | 1.36-4.54 |  |
| Recovery strategies | No | 373(88.6) | 48(11.4) | Ref |  | 0.022 |
|  | Yes | 351(93.3) | 25(6.7) | 1.80 | 1.09-2.99 |  |

Respondents’ details and transport management risk factors for transport pneumonia with a Wald test P value less than 0.250 identified using univariate logistic regression. In the third and fourth column the frequency of the respondent (not reporting and reporting pneumonia) are reported as total number (n) and percentage in each category. Odds ratio (OR); 95% confidence interval (95%CI); ^a^ P value calculated using Wald’s test (P); SB: Standardbred, TB: Thoroughbred; BJ: before journey; AJ: after journey.
